# Cross-Lagged Dyadic Associations of Food Insecurity and Depressive Symptoms among Couples Living with HIV and Cardiometabolic Disorders in Malawi

**DOI:** 10.1016/j.cdnut.2026.109507

**Published:** 2026-08-01

**Authors:** Joshua Kiyingi, Julie T Bidwell, Torsten B Neilands, Sheri D Weiser, Rita M Butterfield, Nancy Mulauzi, James Mkandawire, Amy A Conroy

**Affiliations:** 1 Department of Medicine, Division of Epidemiology, Vanderbilt University Medical Center, Nashville, TN, United States; 2 Caregiving Institute, Betty Irene Moore School of Nursing, University of California Davis, Sacramento, CA, United States; 3 Division of Prevention Science, Department of Medicine, University of California, San Francisco, San Francisco, CA, United States; 4 Division of HIV, Infectious Disease, and Global Medicine, Department of Medicine, University of California, San Francisco, San Francisco, CA, United States; 5 Invest in Knowledge, Zomba, Malawi

**Keywords:** food insecurity, depression, HIV, cardiometabolic disorders, dyadic analysis, Malawi

## Abstract

**Background:**

Household food insecurity is an important social determinant of mental health, human immunodeficiency virus (HIV), and cardiometabolic outcomes in sub-Saharan Africa. Research is limited on how couples managing chronic illness are jointly affected by household food insecurity and depressive symptoms.

**Objectives:**

The aim of our study was to investigate whether household food insecurity predicts subsequent depressive symptoms over time among couples living with HIV and cardiometabolic disorders in Malawi.

**Methods:**

We examined longitudinal dyadic data (4 waves) from 250 couples recruited from HIV and noncommunicable disease clinics in the Zomba and Blantyre districts. Food insecurity was assessed using the 9-item Household Food Insecurity Access Scale, and depressive symptoms were assessed using the Patient Health Questionnaire. Structural equation modeling was used to model actor effects (participant’s food insecurity predicting their own depression) and partner effects (partner’s food insecurity predicting the participant’s depression).

**Results:**

Among females, there were significant actor effects; higher food insecurity at wave 1 predicted greater depressive symptoms at wave 2 (β = 0.162, *P* = 0.022). Among males, there were significant actor effects; higher food insecurity at wave 3 predicted greater depressive symptoms at wave 4 (β = 0.199, *P* = 0.030). Partner effects were not significant at any waves. Household food insecurity predicts future depressive symptoms for males and females, although at different time points. There was no evidence to suggest that one partner’s food insecurity affects the other partner’s depression.

**Conclusions:**

People living with HIV and cardiometabolic disorders may benefit from screening for food insecurity and depressive symptoms and subsequent linkage into services.

## Introduction

Food insecurity, defined as limited or uncertain access to sufficient, safe, and nutritious food for an active, healthy life [1,2], is a growing global public health concern and an influential social determinant of health [3,4]. Severe food insecurity has risen in many low- and middle-income countries, especially in sub-Saharan Africa (SSA), where climate shocks and structural economic vulnerabilities amplify household risk [5]. In 2024, an estimated 8.2% of the global population faced severe food insecurity, compared with >20% in SSA, representing ∼307 million people [6]. Malawi exemplifies this regional burden of food insecurity: nearly 9 million people, almost half the population, experience food insecurity [7,8]. The drivers of food insecurity are multifactorial and include limited income and assets, constrained access to land and agricultural inputs, recurrent droughts and floods, inflation, and unemployment [6,[9], [10], [11], [12], [13]].

Food insecurity adversely affects health through nutritional, psychosocial, and physiological pathways [14,15]. Nutritionally, it leads to inadequate dietary intake and dependence on low-cost, energy-dense foods, reducing essential macronutrient and micronutrient consumption [16]. Psychosocially, the stress and uncertainty of food access can increase depressive symptoms, anxiety, symptoms of posttraumatic stress disorder, and impair mental well-being [[17], [18], [19]]. Physiologically, chronic stress responses can disrupt metabolic and immune regulation, heightening vulnerability to disease [[20], [21], [22]]. Among people living with HIV (PLHIV) in SSA, food insecurity remains highly prevalent and may intensify daily stress, which can worsen mood and coping capacity and undermine adherence to antiretroviral therapy, creating reinforcing pathways between poor nutrition, depression, and HIV disease management [[23], [24], [25], [26], [27]]. Cross-sectional studies conducted in SSA have shown that moderate-to-severe food insecurity is strongly associated with depressive symptoms [28,29].

Most existing studies in SSA have examined the association between food insecurity and depression among individuals, overlooking the household experience of food insecurity and how it impacts family health more broadly. This household perspective is critical because spouses or partners may comanage household resources, make decisions about food allocation and income generation, and support (or strain) one another’s illness management [[30], [31], [32]]. The Dyadic Stress Process Model provides a lens for understanding how partners experience, appraise, and cope with shared stressors [33,34]. It emphasizes that partners’ stress and coping are interdependent, with one partner’s stress potentially affecting the other’s well-being via supportive, shared, or delegated coping [33,34].

Food insecurity may function as a shared stressor; however, it may impact partners differently based on gender roles. Research from Uganda and Kenya shows that females frequently bear responsibility for food preparation and adherence support, whereas males face pressure to provide financially [35,36]. Dyadic research from high-income settings echoes these patterns: financial strain is associated with depressive symptoms in both partners, and one partner’s distress often affects the other [37].

Despite cross-sectional evidence, there is a lack of longitudinal data on how food insecurity and depressive symptoms influence one another, specifically at the household level [25,29]. To fill the gaps in knowledge, we examined temporal links between household food insecurity and depressive symptoms using dyadic data from the Healthy Hearts cohort in Malawi [38]. Couples in the Healthy Hearts study are living with HIV and cardiometabolic disorders (CMDs), hypertension, and diabetes. Thus, within the context of HIV and chronic conditions, we tested whether individual perceptions of household food insecurity predict greater depressive symptoms (actor effects), and whether one partner’s perception of their food insecurity predicts the other partner’s subsequent depressive symptoms (partner effects). We hypothesize that actor effects and partner effects would be positive. By capturing both temporal ordering and dyadic interdependence, this study advances understanding of how food insecurity and mental health interrelate within couples managing chronic illness in a low-resource SSA setting.

## Methods

### Setting

Malawi faces overlapping challenges of poverty, food insecurity, and chronic disease. Nearly half the population, around 9 million people, experience food insecurity, and >4 million face severe hunger [7]. These vulnerabilities are compounded by a high HIV prevalence of 8.9% among adults, a growing burden of CMD such as hypertension (15.8%–32.9%) and diabetes (2.4%–5.6%), and a substantial mental health burden estimated above 20% [[39], [40], [41]]. Poverty is widespread in Malawi, with ∼71% of adults living on <$2.15 USD per day [42]. In Malawi, where this study is conducted, depressive symptoms are prevalent among PLHIV and undermine engagement in care [43].

### Study population and recruitment

The Healthy Hearts study aimed to develop a conceptual model for joint management of HIV and CMD among couples, inform the design of couple-focused interventions in SSA, and improve clinical care and disease management for individuals living with both conditions. The study recruited 250 couples from the Zomba (*n* = 182) and Blantyre (*n* = 68) districts of Malawi. Couples were eligible if they were married or had been cohabiting for ≥6 mo, both partners were aged 18 years or older, and ≥1 partner (the index participant) was living with HIV and either hypertension or diabetes. Partners living with HIV were required to have disclosed their HIV status to their spouse [38]. Research assistants introduced the study during routine morning health talks and invited interested patients to be screened. Index patients were identified in the waiting areas of HIV and noncommunicable disease clinics. Clinic nurses identified new and returning patients with HIV and co-occurring hypertension or diabetes and referred them to the study team. Index patients who met the initial criteria were given an information card to share with their spouse. Research staff then conducted a private telephone screening with the partner to confirm eligibility. Couples in which both partners were eligible and willing to participate were scheduled for a baseline visit. Informed consent was obtained individually and in private from each partner to ensure voluntary participation.

### Data collection and measurements

Data were collected at 4 waves (baseline, 4, 8, and 12 mo) beginning in August 2022. At each wave, both partners were interviewed during the visit but in separate, private rooms by gender-matched interviewers. Interviews were conducted either at participating clinics or at the research center. Trained staff administered standardized, Chichewa-language questionnaires using REDCap on tablets. All 250 couples (500 individuals) completed the baseline assessment. Follow-up rates were high: 240 couples (480 participants; 96%) were reassessed at 4 mo, 237 couples (474 participants; 95%) at 8 mo, and 235 couples (470 participants; 94%) at 12 mo.

### Measures

The outcome measure for this study was depressive symptoms measured using the Patient Health Questionnaire (PHQ-8). The scale consists of 8 questions that assess the frequency and severity of depressive symptoms [44]. This includes items such as “Little interest or pleasure in doing things,” “Feeling down, depressed, or hopeless,” “Trouble falling or staying asleep, or sleeping too much.” Possible responses included not at all = 0, several days = 1, more than half the days = 2, nearly every day = 3. A higher total score (range: 0–24) reflected higher depressive symptoms. This scale has been validated in Malawi [45].

The exposure variable, individual perceptions of household food insecurity, was measured in both partners independently using the 9-items from the Household Food Insecurity Access Scale (HFIAS) [1], which captures household-level experiences with access to food. The scale includes items such as “Did you worry that your household would not have enough food?” and “Did you or any household member have to eat a limited variety of foods, such as only *nsima* (African cornmeal) and vegetables, due to a lack of resources?” Response options included never = 0, rarely = 1, sometimes = 2, and frequently = 3, reflecting the frequency of food insecurity. A total score was calculated from the HFIAS (range: 0–27), with higher scores indicating greater severity of household food insecurity. The scale demonstrated excellent internal consistency (Cronbach’s α = 0.93).

Control variables included age (in years), years of education completed (in years), household wealth, alcohol use, and relationship duration (in years). Household wealth was assessed based on reported assets, using the method proposed by Filmer et al. [46], including ownership of 10 items (bed, television, radio, phone, refrigerator, bicycle, motorcycle, animal-drawn cart, car or truck, and Bible or Koran), access to electricity, water source, type of toilet or latrine, and type of roofing and flooring in the home. Individual items were combined into a wealth score via principal component analysis, which was then divided into wealth quintiles. Alcohol consumption was measured using the Alcohol Use Disorders Identification Test-Consumption [47]. The scale had questions such as “In the past 3 months, how often did you have a drink containing alcohol?” and “How many drinks containing alcohol do you have on a typical day when drinking?” The scale range was 0 to 12, with a higher score indicating greater frequency and quantity of alcohol consumption. The 3-item scale showed acceptable internal consistency (Cronbach’s α = 0.65).

### Statistical analysis

We summarized baseline characteristics for all participants (*n* = 500) using means and SDs for continuous variables and frequencies and percentages for categorical variables. We also present zero-order correlations for food insecurity and depressive symptoms by sex and wave to summarize bivariate associations. To assess temporal and dyadic associations between food insecurity and depressive symptoms, we estimated the cross-lagged actor–partner interdependence model (CL-APIM) [[48], [49], [50]] across 4 waves (baseline, 4, 8, and 12 mo), treating partners as distinguishable dyads based on gender (male/female). Models were fitted in Mplus using robust maximum likelihood and full-information maximum likelihood (FIML) to handle missing data (<3%) under the assumption of missing at random. Time t indexes consecutive waves (1→2, 2→3, 3→4). For each construct (food insecurity and depressive symptoms) and partner (male and female), we included *1*) stability paths, in which depressive symptoms at time t was regressed on depressive symptoms at time t−1 and food insecurity at time t was regressed on food insecurity at time t−1, freely estimated across lags (1→2, 2→3, 3→4), and *2*) bidirectional cross-lagged paths. The cross-lagged structure included actor effects, in which one’s own food insecurity at t−1 predicted one’s own depressive symptoms at t, and partner effects, in which a partner’s food insecurity at t−1 predicted the other partner’s depressive symptoms at t; analogous actor and partner paths were estimated for depressive symptoms at t−1 predicting food insecurity at t. To address potential confounding, every outcome at each time point was regressed on age, years of education completed, relationship duration, household wealth (baseline), and alcohol use from the previous wave (actor and partner). To account for dependence not captured by these predictors, we additionally modeled *1*) within-wave residual covariances and *2*) adjacent-wave residual autocorrelations. Within each wave, we freely estimated residual covariances between depressive symptoms and food insecurity within person, between male and female partners for the same construct (depressive symptoms with depressive symptoms; food insecurity with food insecurity), and across partners and constructs (e.g., male depressive symptoms with female food insecurity, female depressive symptoms with male food insecurity). Model fit was evaluated using root mean square error of approximation (RMSEA) [with 95% confidence interval (CI)], comparative fit index (CFI), and standardized root mean square residual (SRMR), with interpretation based on conventional thresholds [RMSEA ≤0.06 (upper 95% CI ≤ 0.08), CFI ≥0.90 for acceptable and ≥0.95 for good fit, and SRMR ≤0.08 (≤0.05 indicating very good fit)] [51]. Sex differences in actor and partner effects at each lag were assessed using Wald tests comparing male and female paths.

## Results

The mean participant age was 51.1 y (SD = 10.0, range: 20–86), and the mean household size was 4 members. The mean years of education completed was 6.7 (SD = 4.17, range: 0–13), and the mean relationship duration was 21.2 y (SD = 12.32, range: 0.6–54). The mean food insecurity score was 7.49 (SD = 6.85, range: 0–27), and the mean PHQ score was 3.83 (SD = 3.63, range: 0–20). For disease status, 86.2% of the participants were HIV positive, and 46.6% and 16.2% were diagnosed with hypertension and diabetes, respectively (Table 1). In the zero-order correlations for food insecurity and depressive symptoms by sex and wave, variables displayed moderate stability; concurrent within-person food insecurity and depressive symptoms correlations were positive across all waves; cross-partner correlations were smaller (Table 2).

**TABLE 1:** Baseline characteristics of healthy hearts participants (*N* = 500/couples = 250)

Variable	Total sample (*N* = 500) *n* (%)/mean (SD)	Male (*N* = 250) *n* (%)/mean (SD)	Female (*N* = 2 50) *n* (%)/mean (SD)
Age (y) (min/max: 20–86)	51.07 (9.96)	54.93 (9.62)	47.21 (8.75)
Years of education completed (min/max: 0–13)	6.65 (4.17)	7.38 (4.15)	5.91 (4.07)
Relationship duration in years	21.17 (12.12)	21.17 (12.12)	21.17 (12.12)
Household size (min/max: 2–10)	4.00 (1.50)	4.00 (1.50)	4.00 (1.50)
Household wealth	0.00 (1.77)	0.11 (1.79)	0.11(1.74)
Alcohol use (AUDIT-C) (min/max: 0–12)	0.56 (1.55)	1.04 (2.03)	0.08 (2.03)
Household food insecurity (min/max: 0–27)	7.49 (6.85)	6.16 (6.63)	8.82 (6.82)
Depressive symptoms (min/max: 0–20)	3.83 (3.63)	2.73 (6.82)	4.93 (4.13)
Disease status			
HIV	431 (86.20)	209 (83.60)	222 (88.80)
Hypertension	233 (46.60)	115 (46.00)	118 (47.20)
Diabetes	81 (16.20)	25 (10.00)	56 (22.40)

Abbreviation: AUDIT-C, Alcohol Use Disorders Identification Test-Consumption.

**TABLE 2:** Pearson correlations between food insecurity and depressive symptoms by sex and wave

	Variables	1	2	3	4	5	6	7	8	9	10	11	12	13	14	15	16
1	PHQ_M1	1															
2	PHQ_F1	0.055	1														
3	FI_M1	0.298∗∗∗	0.145∗∗	1													
4	FI_F1	0.044	0.355∗∗∗	0.450∗∗∗	1												
5	PHQ_M2	0.444∗∗∗	0.109∗	0.322∗∗∗	0.171∗∗∗	1											
6	PHQ_F2	0.035	0.373∗∗∗	−0.013	0.149∗∗	0.012	1										
7	FI_M2	0.056	0.043	0.534∗∗∗	0.292∗∗∗	0.230∗∗∗	−0.072	1									
8	FI_F2	−0.028	0.175∗∗∗	0.317∗∗∗	0.540∗∗∗	0.095∗	0.241∗∗∗	0.326∗∗∗	1								
9	PHQ_M3	0.282∗∗∗	0.104∗	0.310∗∗∗	0.141∗∗	0.419∗∗∗	0.087	0.239∗∗∗	0.043	1							
10	PHQ_F3	−0.023	0.176∗∗∗	−0.097∗	0.046	0.084	0.390∗∗∗	−0.143∗∗	0.086	−0.03	1						
11	FI_M3	0.180∗∗∗	0.099∗	0.614∗∗∗	0.373∗∗∗	0.319∗∗∗	−0.158∗∗∗	0.627∗∗∗	0.201∗∗∗	0.342∗∗∗	−0.179∗∗∗	1					
12	FI_F3	0.04	0.129∗∗	0.08	0.374∗∗∗	0.150∗∗∗	0.294∗∗∗	0.073	0.508∗∗∗	0.048	0.417∗∗∗	0.018	1				
13	PHQ_M4	0.324∗∗∗	0.029	0.304∗∗∗	0.117∗	0.446∗∗∗	0.008	0.214∗∗∗	0.003	0.547∗∗∗	−0.035	0.348∗∗∗	0.054	1			
14	PHQ_F4	0.013	0.243∗∗∗	−0.054	0.025	0.116∗	0.239∗∗∗	0.023	0.086	0.165∗∗∗	0.235∗∗∗	0.004	0.178∗∗∗	0.051	1		
15	FI_M4	0.079	0	0.523∗∗∗	0.279∗∗∗	0.274∗∗∗	−0.110∗	0.621∗∗∗	0.214∗∗∗	0.229∗∗∗	−0.128∗∗	0.666∗∗∗	0.05	0.287∗∗∗	−0.03	1	
16	FI_F4	−0.04	0.122∗∗	0.193∗∗∗	0.238∗∗∗	0.095∗	−0.023	0.298∗∗∗	0.330∗∗∗	0.149∗∗	0.089	0.230∗∗∗	0.372∗∗∗	0.068	0.347∗∗∗	0.341∗∗∗	1

*P* < 0.10; ∗*P* < 0.05; ∗∗*P* < 0.01; ∗∗∗*P* < 0.001 (2-tailed). Ns range: 235–250 couples.

Abbreviations: F, female; FI, food insecurity; M, male; PHQ, Patient Health Questionnaire; 1–4: waves.

**FIGURE 1:**
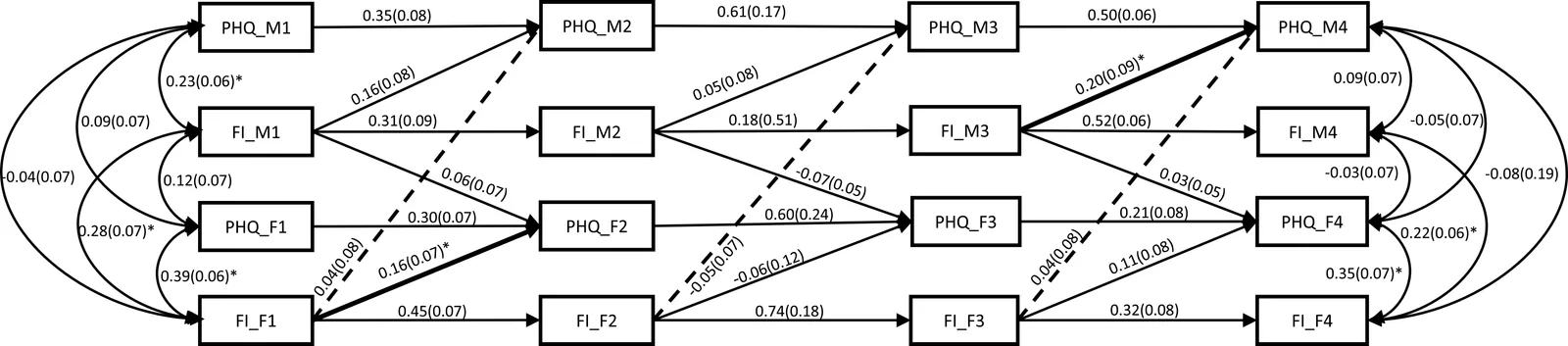
Cross-lagged actor and partner effects of household food insecurity on subsequent depressive symptoms. Some residual correlations (e.g., at waves 2 and 3) are not displayed to maximize clarity and readability. *N* = 250 couples. The figures show β (SE). Solid line = actor FI →PHQ; dashed line = partner FI →PHQ; thicker lines with “∗” = *P* < 0.05. Thin lines = stability path. F, female; FI, food insecurity; M, male; 1–4, waves; PHQ, Patient Health Questionnaire.

The CL-APIM model fit was good: RMSEA = 0.051 (90% CI: 0.040, 0.063), CFI = 0.931, and SRMR = 0.036, as presented in Table 3 and Figure 1. After controlling for covariates described above, the analysis revealed significant actor effects of food insecurity on depressive symptoms among both males and females. For females, higher food insecurity at wave 1 predicted higher change in depressive symptoms from wave 1 to wave 2 (β = 0.162, SE = 0.071, *P* = 0.022); effects at 2→3 (β = −0.059, SE = 0.119, *P* = 0.616) and effects at 3→4 (β = 0.106, SE = 0.079, *P* = 0.177) were not significant. For males, food insecurity at wave 3 predicted higher change in depressive symptoms from wave 3 to wave 4 (β = 0.199, SE = 0.091, *P* = 0.030); effects at 1→2 (β = 0.162, SE = 0.084, *P* = 0.054) and effects at 2→3 (β = 0.054, SE = 0.077, *P* = 0.477) were not significant. Partner effects on depressive symptoms were not significant for either males or females.

**TABLE 3:** Cross-lagged actor and partner effects of household food insecurity on subsequent depressive symptoms (*N* = 500/couples = 250)

Outcome	Lag	Actor FI(*t* − 1) → own PHQ(*t*) β (SE)	*P* value	Partner FI(*t* − 1) → partner PHQ(*t*) β (SE)	*P* value
Male	1→2	0.162 (0.084)	0.054	0.063 (0.067)	0.348
Male	2→3	0.054 (0.077)	0.477	−0.071 (0.054)	0.190
Male	3→4∗	0.199 (0.091)∗	0.030∗	0.026 (0.047)	0.580
Female	1→2∗	0.162 (0.071)∗	0.022∗	0.037 (0.082)	0.651
Female	2→3	−0.059 (0.119)	0.616	−0.054 (0.067)	0.416
Female	3→4	0.106 (0.079)	0.177	0.043 (0.083)	0.604

*χ*^2^(df) = 260.03 (160)∗, RMSEA (90% CI): 0.051 (0.040–0.063), CFI: 0931, TLI: 0.866, SRMR: 0.036.

Abbreviations: CFI, comparative fit index; CI, confidence interval; FI, food insecurity; PHQ, Patient Health Questionnaire; RMSEA, root mean square error of approximation; SRMR, standardized root mean square residual; *t*, wave; TLI, Tucker-Lewis Index

∗ Significant at *P* = 0.05. We adjusted for age, years of education, relationship duration, household size, household wealth, and alcohol use.

## Discussion

This longitudinal, dyadic study examined whether household food insecurity predicts subsequent depressive symptoms among couples living with HIV and CMD in Malawi. Using a CL-APIM with longitudinal data from 4 waves, we found significant actor effects: higher food insecurity predicted greater depressive symptoms at the next wave. These effects were time-specific and differed by sex, with females showing an earlier association (wave 1–2) and males a later association (wave 3–4). We did not detect any lagged partner effects, whereby one partner’s food insecurity predicted the other partner’s later depressive symptoms.

These findings align with evidence from SSA linking food insecurity to poorer mental health, including higher depressive symptom burden [52,53]. The actor effect among females is consistent with gendered caregiving roles: females often shoulder responsibility for ensuring household food provision while having limited control over financial resources, which can intensify worry, guilt, and self-blame when food is scarce [28,54,55]. For males, a significant actor effect may reflect cumulative strain; depressive symptoms may emerge when prolonged scarcity threatens provider-role identity and social norms that discourage early disclosure of distress among males may also delay observed associations [[56], [57], [58]]. This mechanism could also explain the greater magnitude of association observed (food insecurity and depression) for males than for females. Taken together, these patterns suggest that males and females experience the same impacts of food insecurity and depression but not necessarily at the same time.

We interpreted our results through dyadic perspectives on stress and coping. Economic pressure, manifested as food insecurity, appears to increase individual distress through actor pathways, whereas the absence of a lagged partner effect in depressive symptoms suggests that couples in this setting may primarily cope with scarcity in individualized ways [30,31,32]. Several mechanisms could contribute to the null partner effects. Food insecurity in this study was measured as each individual’s perception of their household’s food security rather than as a single shared household-level indicator. Although partners lived in the same household, their experiences of food access may differ substantially because of intrahousehold resource allocation. Intrahousehold resource allocation in Malawi often reflects gender-based differences (for instance, females may eat last and receive smaller portions), resulting in unequal exposure to food scarcity among household members, even when the household is broadly categorized as food insecure. Second, social norms may promote individualized coping; females may minimize distress to avoid worrying their partner or disrupting caregiving; males may downplay depressive symptoms due to masculinity [59,60]. Third, in households managing HIV and CMD, partner effects may operate through other dyadic outcomes (for example, adherence, conflict, or relationship quality) rather than depressive symptoms per se or may manifest simultaneously rather than with a 1-wave lag.

### Strengths and limitations

This study has several important strengths. We leveraged 4 waves of dyadic data with high retention [because of proactive tracking of participants, low participant burden (fewer and shorter visits), and flexible scheduling of follow-up visits], modeled distinguishable partners, and specified a rigorous CL-APIM that accounted for prior symptoms, correlated partner outcomes, and key covariates, using robust estimation and FIML for missing data. These design and analytic features improve temporal inference relative to cross-sectional studies and allow nuanced interpretation of actor vs. partner processes. The study also has limitations, including the use of a nonrepresentative sample. Specifically, the study focused on middle-aged couples with chronic illnesses, which may limit the generalizability of the findings to the broader population in Malawi. Future research should consider including younger couples and same-sex couples to enhance the generalizability of the results.

### Study implications

Our findings are important, as both food insecurity and mental health are linked to reduced ability to engage in self-management of chronic illnesses such as HIV and CMD [61]. The findings also have syndemic implications because food insecurity, HIV and other chronic illnesses, and poor mental health may cluster and mutually reinforce one another. Consistent with findings from Ghana that showed persistent household food insecurity and HIV were independently associated with maternal stress, whereas their co-occurrence was associated with substantially greater stress. In the present context, this suggests that food insecurity should not be treated as an isolated social determinant but as part of an interconnected burden involving HIV, CMD, and poor mental health [27]. To address the mental health impacts of food insecurity in HIV and CMD care, food support should be integrated with routine mental health screening and treatment [23,24,25]. Effective options include clinic-linked food vouchers or cash transfers, referrals to social protection, and scalable livelihood and psychological interventions. Couple-focused strategies, such as economic empowerment and relationship strengthening, have shown promise in Malawi and could be adapted to address gendered stressors, including females’ caregiving burdens and male’s provider role pressures [21]. Interventions could potentially be made more impactful by incorporating joint financial planning, communal coping, and communication skills to support collaborative responses to food insecurity [[30], [31], [32]]. At the policy level, efforts to stabilize food prices, expand social protection, and invest in climate-resilient agriculture can reinforce clinic-based strategies [6,11]. Future research should strengthen causal inference using random-intercept CL-APIM or similar within-person models and test longitudinal and cross-sex measurement invariance.

## Conclusions

Food insecurity prospectively predicts depressive symptoms within individuals in Malawian couples managing HIV and cardiometabolic disease, with timing and magnitude varying by sex. The absence of detectable lagged partner effects suggests that individualized internalization of shared economic stress is likely, potentially shaped by gendered roles and coping norms. Addressing both food insecurity and mental health, attuned to dyadic realities, may be essential to improve well-being and clinical outcomes in similar low-resource settings.

## Author contributions

The author’s responsibilities were as follows – AAC: conceptualized, obtained funding, supervised the study, contributed to all stages of data analysis, and wrote the manuscript; JK: analyzed participant data and wrote the manuscript; JTB, SDW: conceptualized the study and reviewed and edited the manuscript; TBN: contributed to data analysis; RMB: curated the data and administered the project; NM, JM: administered project and collected data; AAC, TBN, JTB, RMB, SDW, NM, JM: reviewed and edited the manuscript; and all authors: read and approved the final manuscript.

## Data availability

The datasets generated and/or analyzed during the current study are not publicly available due to the sensitivity of the data but are available from the corresponding author on reasonable request.

## Ethical approval

This study was conducted in accordance with the guidelines outlined in the Declaration of Helsinki, and all procedures involving research study participants were approved by the Human Research Protection Program at the University of California, San Francisco, and the National Health Science Research Committee in Malawi. Written informed consent was obtained from all subjects/patients.

## Declaration of Generative AI and AI-assisted technologies in the writing process

The author(s) declare that no generative AI or AI-assisted technologies were used in the writing of this manuscript.

## Funding

This work was supported by the NIH under grant number R01-HL153343. The funder had no role in the study design, data collection, analysis, interpretation, article writing, or the decision to submit the article for publication.

## Conflict of interest

AAC reports financial support was provided by NIH. All other authors report no conflicts of interest.
